# Cytotoxic, Antioxidant, and Anti-Genotoxic Properties of Combretastatin A4 in Human Peripheral Blood Mononuclear Cells: A Comprehensive In Vitro Study

**DOI:** 10.3390/biom14121535

**Published:** 2024-11-30

**Authors:** Petar Popović, Andrea Pirković, Dijana Topalović, Lada Živković, Milica Marković, Biljana Spremo-Potparević

**Affiliations:** 1 Department of Pathobiology, Faculty of Pharmacy, University of Belgrade, 11000 Belgrade, Serbia; petar.popovic@pharmacy.bg.ac.rs (P.P.); dijana.topalovic@pharmacy.bg.ac.rs (D.T.); lada.zivkovic@pharmacy.bg.ac.rs (L.Ž.); mmarkovic@pharmacy.bg.ac.rs (M.M.); biljana.potparevic@pharmacy.bg.ac.rs (B.S.-P.); 2 Department for Biology of Reproduction, Institute for the Application of Nuclear Energy-INEP, University of Belgrade, 11000 Belgrade, Serbia

**Keywords:** combretastatin A4, PBMC, oxidative stress, genotoxicity, trophoblast

## Abstract

Despite significant advances in drug discovery and the promising antitumor potential of combretastatin A4 (CA-4), which selectively targets rapidly dividing cancer cells, CA-4’s effects on non-dividing human cells, such as peripheral blood mononuclear cells (PBMCs), remain unclear. The aim of this study is to evaluate the in vitro bioactivity of CA-4 in human PBMCs, focusing on its antigenotoxic and antioxidant properties, while comparing its cytotoxic potency against PBMCs, cancer cell lines (JAR and HeLa), and the normal trophoblast cell line HTR-8/SVneo. Cell viability and metabolic activity were evaluated using the MTT assay. ROS production in PBMCs was measured using the H2DCFDA assay, and DNA damage was assessed using the Comet assay. CA-4 showed cytotoxicity in PBMCs and HTR-8/SVneo cells at concentrations above 200 µM, while cancer cells, JAR and HeLa, showed cytotoxicity at 100 µM and 1 µM, respectively. CA-4 also reduced ROS levels in PBMCs under oxidative stress and showed antioxidant effects at concentrations from 1 to 200 µM. In addition, CA-4 showed antigenotoxic effects against H_2_O_2_-induced DNA damage in PBMCs at concentrations of up to 1 µM. CA-4 exhibited lower cytotoxicity in human PBMCs compared to cancer cells, inhibited ROS production, and showed antioxidant and antigenotoxic properties, providing insight into its potential therapeutic efficacy and safety.

## 1. Introduction

One of the biggest challenges in cancer treatment is the uncontrolled proliferation of tumor cells and the need for more selective therapies. Conventional cancer therapies are often limited by toxicity and the development of drug resistance, partly due to the complexity of the tumor microenvironment. This underscores the importance of ongoing research to discover or develop new agents that can improve therapeutic efficacy while reducing adverse effects. In the past, many effective drugs have been derived from natural sources and subsequently optimized for improved efficacy [1].

Combretastatin A4 (CA-4), a plant-derived compound isolated from African willow (*Combretum caffrum* (Eckl. & Zeyh.) Kuntze) in 1989 [2], belongs to a family of structurally related compounds known for their potent antimitotic and antiproliferative activities [3,4,5]. These compounds include stilbenes (combretastatin A), dihydrostilbenes (combretastatin B), phenanthrenes (combretastatin C), and macrocyclic lactones (combretastatin D) [4]. Their biological activity is closely related to their stereoisomeric configurations, and both natural and synthetic derivatives are of great interest in clinical trials for cancer chemotherapy [6,7,8]. Remarkably, despite their name, the combretastatins are not structurally related to the statins.

In clinical studies of patients with advanced solid tumors, CA-4 has demonstrated a “vascularly active” profile with minimal cytotoxic side effects [7,8]. Early studies in mouse models of melanoma, lung, ovarian, and colon cancer showed that the anticancer effect of CA-4 is primarily due to inhibition of angiogenesis, reduction of blood flow to tumor tissue, and damage to the endothelium of blood vessels supplying the tumor [5,9]. Mechanistically, CA-4 binds to the colchicine-binding site on tubulin, inhibits its polymerization, disrupts the structural integrity of the cell, and arrests the cell cycle in the G2/M phase. This leads to mitotic catastrophe and activation of apoptosis pathways, ultimately resulting in cell death [10,11].

In addition, CA-4 has been investigated for its effects on the production of reactive oxygen species (ROS) in cancer cells [6,12,13]. Cancer cells typically have higher baseline levels of ROS than normal cells due to their altered metabolism and rapid proliferation [14,15]. CA-4 further increases ROS levels and exacerbates oxidative stress in cancer cells. This excessive ROS production leads to oxidative damage that disrupts important cellular functions and leads to cell death. Interestingly, although CA-4 disrupts antioxidant defenses in cancer cells, it has also been reported to exhibit potent antioxidant properties compared to other analogous compounds [4,16].

CA-4 can also exacerbate DNA damage in cancer cells, and this exacerbated DNA damage, in conjunction with their impaired DNA repair mechanisms, increases their susceptibility to apoptosis [17,18]. Single-strand breaks (SSBs) are the most common form of DNA damage, occurring approximately 10,000 times per day by endogenous ROS. Normally, these breaks are efficiently repaired by the base excision repair (BER) mechanism. However, if left unrepaired, especially in proliferating cells, SSBs can lead to replication fork collapse, resulting in double-strand breaks (DSBs)—the most lethal form of DNA damage [19]. In many cancers, defective DNA repair mechanisms increase the baseline level of DNA damage [20]. By exacerbating this stress, CA-4 increases DNA damage beyond the cells’ ability to repair, eventually exceeding their homeostatic threshold and leading to cell death [18].

Lymphocytes, which make up to 20–40% of leukocytes, play a central role in the adaptive immune response. They defend the body against pathogens and eliminate abnormal cells, including tumor cells. Healthy lymphocytes are critical for effective immune surveillance, a process in which the immune system continuously monitors the body for cancer cells. Important subsets of lymphocytes, such as cytotoxic T cells and natural killer (NK) cells, contribute directly to the elimination of tumor cells. Cytotoxic T cells recognize specific antigens on tumor cells and initiate their destruction, while NK cells attack tumor cells that evade the adaptive immune system. Without a robust population of healthy lymphocytes, the immune system’s ability to control tumor growth is impaired, increasing the risk of cancer progression and metastasis [21,22].

The aim of this study was to evaluate the *in vitro* effects of CA-4 on the survival and function of healthy human peripheral blood mononuclear cells (PBMCs), primarily lymphocytes, focusing on its potential cytotoxic, antioxidant, and antigenotoxic properties. In addition, for the first time, the cytotoxic effect of CA-4 was investigated in both normal human trophoblast cells and malignant trophoblast cells (choriocarcinoma cells). Given the critical role of trophoblast cells in pregnancy, understanding the safety profile of CA-4 in these cells is crucial for assessing its potential risks during pregnancy and, thus, expanding our understanding of its broader effects. The HeLa cell line was used as a reference cancer cell line, which is the most commonly used model for studying human cancer biology.

## 2. Materials and Methods

### 2.1. Treatment Preparation

For cytotoxic evaluation, a stock solution was prepared by dissolving CA-4 in DMSO (Sigma Aldrich, St. Louis, MO, USA) at a concentration of 100 mM. For the experiments, serial dilutions were prepared from the stock solution, and control cells were exposed to an equivalent concentration of DMSO (no more than 0.2% at the highest treatment concentration). 

### 2.2. Subjects and PBMC Isolation

Peripheral blood samples from four healthy donors (two males and two females) who reported no drug, cigarette, or alcohol use or medical therapy prior to the study were collected in heparinized containers. The participants gave informed consent in accordance with the regulations of the Ethics Committee for Clinical Trials of the University of Belgrade—Faculty of Pharmacy.

Samples were collected in vacutainer tubes with lithium heparin (Vacuette, Greiner Bio-One, Kremsmuenster, Austria). A total of 5 mL of blood was collected per participant for the analyses, and the PBMCs were separated immediately. For the separation of PBMCs on Ficoll-Paque medium (GE Healthcare Bio-Sciences, Uppsala, Sweden), a volume of 4 mL of anticoagulant blood was used and centrifuged at 450× *g* for 15 min. The PBMCs in the annular layer formed directly above the Ficoll-Paque medium were collected with a pipette and washed twice, first in phosphate-buffered saline (PBS, Fisher Scientific, Pittsburgh, PA, USA) and then in RPMI 1640 medium (Gibco, Waltham, MA, USA). Each wash was followed by centrifugation at 300× *g* for 5 min. After the final wash and centrifugation, the supernatant was carefully removed so as not to disturb the PBMC pellet at the bottom of the tube. The pellet was suspended in 1 mL of complete RPMI medium (RPMI 1640 medium + 10% fetal calf serum (FCS, Pan Biotech, Aidenbach, Germany) + 1% penicillin/streptomycin (Capricorn Scientific GmbH, Ebsdorfergrund, Germany)) by careful mixing with a pipette and used immediately for further analysis.

### 2.3. Cytotoxic Activity

The cytotoxic effect of CA-4 was investigated on human PBMCs, as well as on HTR-8/SVneo (human trophoblast), JAR (human choriocarcinoma), and HeLa (human cervical carcinoma) cells, by MTT assay. 

### 2.4. Cell Culture

Three different cell lines, one non-malignant and two cancer cell lines, were used to evaluate the cytotoxicity of combretastatin A4. The non-malignant HTR-8/SVneo extravillous trophoblast cell line was derived from human explant cultures of first-trimester placenta immortalized by SV40 large T antigen, which was provided courtesy of Dr. Charles H. Graham, Queen’s University, Kingston, Canada. Cells were cultured in complete RPMI medium. The JAR choriocarcinoma cell line (ATCC^®^, American Type Culture Collection, VA, USA) and human cervical adenocarcinoma (HeLa, ATCC^®^ CCL-2™, American Type Culture Collection, VA, USA) were also cultured in complete RPMI medium. All cell lines were grown in 25 cm^2^ tissue-culture flasks and maintained at 37 °C, 5% CO_2_, in a humidified incubator. 

### 2.5. MTT Assay

After reaching 70% confluence, the cells were harvested from the flasks with a 0.25% trypsin–EDTA solution (Institute of Virology, Vaccines and Serum “Torlak”, Belgrade, Serbia) and seeded in 96-well plates (2 × 10^4^ cells/well for HTR-8/SVneo and JAR and 1.5 × 10^4^ cells/well for HeLa cells) in 100 µL of complete medium and allowed to adhere to the plates for 24 h. The next day, the medium was removed, and fresh control medium or the complete medium containing the CA-4 treatments was added in a total culture volume of 100 µL per well. The cells were incubated with the treatments for 24 h. For PBMC analysis, the isolated cells were seeded at 3 × 10^5^ cells/well in 96-well plates and incubated for 24 h at 37 °C with CA-4 in complete medium. After incubation, the medium was replaced with 100 μL/well of fresh complete medium containing thiazolyl blue tetrazolium bromide (MTT) at a final concentration of 0.5 mg/mL (Acros Organics, Fisher Scientific Company, Fair Lawn, NJ, USA), and the cells were left in the dark at 37 °C for 4 h. The formed formazan crystals were further dissolved by adding sodium dodecyl sulfate (10% SDS in 0.01 M HCl, Sigma Aldrich, St. Louis, MO, USA) at 100 µL per well and left at 37 °C for 24 h. The next day, the plates were shaken for 5 min, and the absorbance was measured at 570 nm, using a microplate reader (BioTek ELx800, Thermo Fisher Scientific, MA, USA). Each experiment was performed three times in triplicate. Cell viability was expressed as the percentage of viable cells compared to the control. The viability assay data were also used to calculate the IC_50_ value, which is the half-maximal inhibitory concentration or the concentration that results in a 50% reduction in cell viability. The values were expressed in comparison to the reference substance, cisplatin.

### 2.6. Cellular Reactive Oxygen Species Production—H2DCFDA Assay 

Isolated PBMCs were seeded in complete RPMI medium in 96-well plates at a density of 3 × 10^5^ cells per well, in a final volume of 100 µL per well, and CA-4 treatments at final concentrations in complete medium were added to the cells. After 24 h, the treatments were removed, and the cells were then washed with PBS. The H2DCFDA (2′,7′-dichlorofluorescin diacetate) assay was then performed according to the manufacturer’s instructions. Using PBS as a solvent, a solution of 5 µM H2DCFDA (CAS 4091-99-0, Merck Millipore Calbiochem, St. Louis, MO, USA), which is sensitive to oxidation in cells, was added to each well and left in the dark for 45 min, until the reaction occurred. Then, the cells were washed with PBS and treated with PBS alone (control) or 50 µM H_2_O_2_ to induce ROS production in the cells. After incubation for 30 min and conversion of H2DCFDA to highly fluorescent 2′,7′-dichlorofluorescin (DCF), the generation of intracellular ROS in the cells was determined by measuring fluorescence, using a plate reader (Wallac 1420 Multilabel Counter Victor 3V, Perkin Elmer, MA, USA), at wavelengths of 485–535 nm. The data are expressed as relative fluorescence intensity, which is directly proportional to the amount of ROS. Analysis of four independent samples of isolated cells from volunteers was performed in triplicate (n = 12).

### 2.7. The Alkaline Comet Assay

The extent of DNA damage was determined using the alkaline comet assay. For the treatments, a stock solution was prepared by dissolving CA-4 in DMSO at a concentration of 2 mM, and serial dilutions with PBS were prepared from the stock solution for the experiments. The suspension of PBMCs in low-melting-point agarose (Sigma-Aldrich, St. Louis, MO, USA) was applied to slides previously coated with normal-melting-point agarose (Sigma-Aldrich, St. Louis, MO, USA). 

The genotoxic potential was determined by incubating the cells for 1 h at 37 °C with the different CA-4 concentrations and comparing the observed effects with controls treated with solvent alone. Antigenotoxic activity was analyzed in the pre- and post-treatment protocols. For pre-treatment, cell preparations were treated with CA-4 for 30 min at 37 °C and rinsed with PBS before 50 µM of hydrogen peroxide (H_2_O_2_) was added at 4 °C for 20 min. For post-treatment, cells were subjected to the same treatments in reverse order.

After the treatments, the slides were immersed in cold lysis solution containing 100 mM EDTA (Sigma-Aldrich, St. Louis, MO, USA), 2.5 M NaCl (T.T.T. doo., Sveta Nedelja, Croatia), 10 mM Tris (Sigma-Aldrich, St. Louis, MO, USA), 1% Triton × 100 (Fisher Scientific, Pittsburgh, PA, USA), and 10% dimethyl sulfoxide (Fisher Scientific, Pittsburgh, PA, USA); adjusted to a pH of 10 with NaOH (Fisher Scientific, Pittsburgh, PA, USA); and then left overnight at 4 °C. The next day, the slides were immersed in the cold alkaline electrophoresis buffer (10 M NaOH, 200 mM EDTA, pH 13) for 30 min. Electrophoresis was performed at 0.8 V/cm for 30 min in a horizontal gel electrophoresis tank (CHU2, Scie-Plas Ltd., Cambridge, UK), connected to a Power Supplier EPS 601 (GE Healthcare, Amersham, UK). After electrophoresis, neutralization was performed twice for 10 min with neutralization buffer (0.4 M Tris base, pH 7.5 adjusted with HCl) and once with distilled water. Slides were stained with ethidium bromide (20 µg/mL), and the degree of DNA damage was assessed via visual classification. The analysis was performed with a fluorescence microscope (Olympus BX 50, Olympus Optical Co., GmbH, Hamburg, Germany) equipped with an HBO mercury lamp (50W, 516-560 nm, Zeiss). To determine the degree of DNA damage, a 100 randomly selected nucleoids from each duplicate slide were analyzed according to Collins et al. [23], assigning each of the comets a value between 0 and 4 (from 0, undamaged, to 4, completely damaged). The degree of DNA damage was calculated as the Total Comet Score (TCS) by applying the following formula for the TCS: 0n +1n +2n +3n +4n, where “n” represents the number of cells in each class [24]. All experiments were performed three times, each time in duplicate.

### 2.8. Statistical Analysis

A one-way analysis of variance (ANOVA) with Tukey post hoc analysis was used to assess the differences between the treatments and the control after testing the data for normality. All results are expressed as mean + standard error of the mean (mean + S.E.M). GraphPad Prism 6.0 (GraphPad Software, Inc., version 8.4.3.686, La Jolla, CA, USA) was used for statistical analysis, and *p* < 0.05 was considered significant.

## 3. Results

Given the current understanding of the bioactivity and efficacy of CA-4, our research aimed to investigate its potential cytotoxic, antioxidant, and antigenotoxic effects on human PBMCs. We evaluated its selectivity by comparing its cytotoxic potency against healthy PBMCs and trophoblast cells (HTR-8/SVneo) with tumor cell lines (JAR and HeLa).

**Figure 1:**
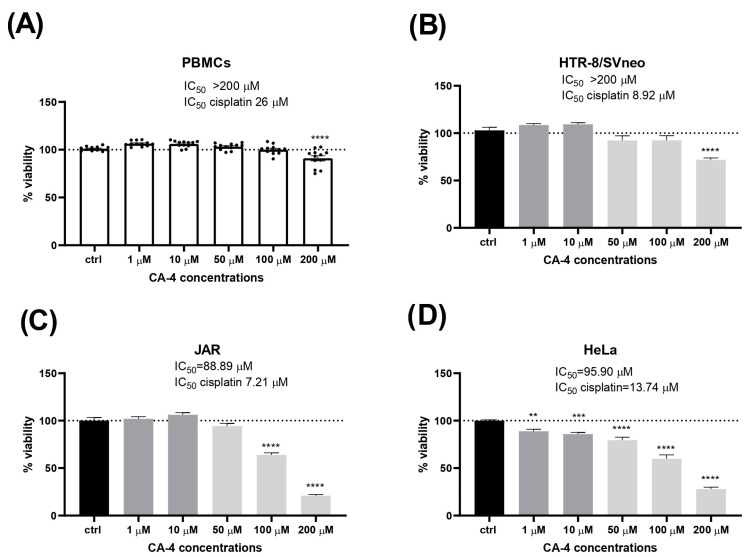
Cytotoxic effects of combretastatin A4 (CA-4): Effect of CA-4 on viability and cytotoxicity of isolated human primary peripheral blood lymphocytes (PBMCs) (**A**), normal human trophoblast cells HTR-8/SVneo (**B**), and cancer cells JAR (**C**) and HeLa (**D**) after 24 h of treatment with a range of concentrations (1–200 µM). Cell viability was determined using the MTT assay. Results are expressed as mean + S.E.M. ** *p* < 0.01, *** *p* < 0.001, and **** *p* < 0.0001 vs. ctrl.

First, we investigated the influence of CA-4 on the survival and metabolic activity of freshly isolated normal human PBMCs from healthy donors. The degree of reduction in the MTT reagent reflects the metabolic activity, which correlates with the number of viable cells. As shown in Figure 1, CA-4 at concentrations below 200 µM showed no statistically significant effect on viability or metabolic activity compared to untreated control cells. A significant decrease in metabolic activity was only observed at the highest concentration (200 µM), while lower concentrations (1, 10, 50, and 100 µM) either maintained or slightly increased metabolic activity. Similarly, normal human trophoblast cells (HTR-8/SVneo) tolerated CA-4 exposure up to 100 µM without significant effects on viability, with a marked decrease at 200 µM (IC_50_ of CA-4 > 200 µM vs. IC_50_ of cisplatin 8.92). Of note, the HTR-8/SVneo trophoblast cells exhibited differential sensitivity to CA-4 compared to the malignant cell lines, as shown in Figure 1.

CA-4 showed moderate cytotoxicity in both HeLa (IC_50_ of CA-4 95.90 versus IC_50_ of cisplatin 13.74) and JAR cells (IC_50_ of CA-4 88.89 versus IC_50_ of cisplatin 7.21) compared to the reference compound, cisplatin. In JAR choriocarcinoma cells, a significant reduction in cell viability was observed with 100 µM CA-4. Only the two highest concentrations of CA-4 significantly reduced viability in the JAR cell line. In cervical cancer cells (HeLa), CA-4 decreased viability in a concentration-dependent manner, with the significant decrease starting at 1 µM.

**Figure 2:**
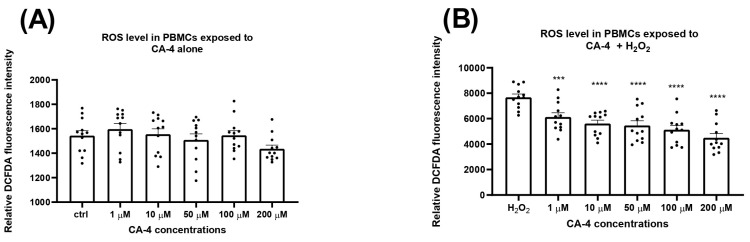
Antioxidant effect of combretastatin A4 (CA-4): The effect of CA-4 on the production of ROS in cells of isolated primary human peripheral blood lymphocytes (PBMCs) after 24 h of treatment with a range of concentrations (1–200 µM), without exposure to hydrogen peroxide (H_2_O_2_) (**A**) and in cells treated with H_2_O_2_ (**B**). The relative fluorescence intensity is directly proportional to the production of ROS in the cells. Data are expressed as mean + S.E.M. *** *p* < 0.001, and **** *p* < 0.0001 vs. H_2_O_2_.

In the next phase of this study, we investigated the effects of CA-4 on reactive oxygen species (ROS) production in isolated PBMCs after 24 h of treatment with concentrations ranging from 1 to 200 µM. Figure 2A shows the fluorescence intensity, representing spontaneous ROS production without hydrogen peroxide exposure. No statistically significant differences were observed between the groups treated with different concentrations of CA-4. However, when cells were exposed to oxidative stress (Figure 2B) by hydrogen peroxide, a statistically significant, concentration-dependent decrease in fluorescence intensity was observed, indicating a corresponding decrease in ROS concentration. These results suggest that CA-4 exerts an antioxidant and protective effect on healthy lymphocytes.

**Figure 3:**
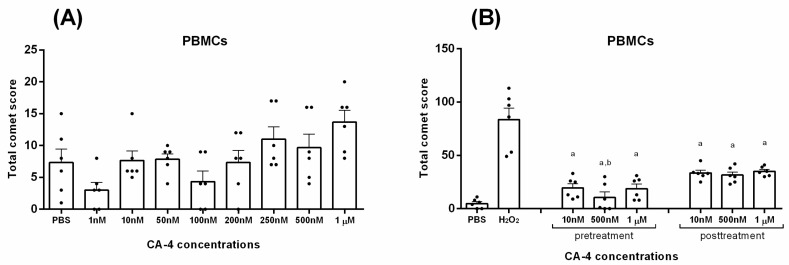
Genotoxic and antigenotoxic effects of combretastatin A4 (CA-4): The effect of CA-4 on the extent of DNA damage in cells of isolated primary human PBMCs after 30 min treatment with a range of concentrations (1 nM–1 µM) (**A**) and the effect of three concentrations (10 nM, 500 nM and 1 µM) on the extent of DNA damage in cells of isolated primary human PBMCs at 30 min pre- and post-treatment (before/after exposure to H_2_O_2_). The extent of DNA damage is calculated as Total Comet Score (TCS), according to the following formula: 0n +1n +2n +3n +4n (n—number of cells in each class). And the results are expressed as mean + S.E.M. a is *p* < 0.0001 vs. H_2_O_2_, and b *p* < 0.05 vs. 1 µM and 10 nM in post-treatment (**B**).

Finally, considering the different effects that combretastatins might have on DNA damage in normal cells compared to cancer cells, we investigated the effects of CA-4 on DNA damage in PBMCs. DNA damage was assessed using the alkaline comet assay, and the results were expressed as the Total Comet Score (TCS), as described in the Materials and Methods section. As shown in Figure 3A, no significant increase in TCS was observed after 30 min of exposure of PBMCs to CA-4 compared to solvent-only controls. Interestingly, in contrast to its pro-damaging effect on cancer cells, CA-4 showed a significant reduction in DNA damage in PBMCs under oxidative stress conditions. The antigenotoxic effect of CA-4 in PBMCs is shown in Figure 3B. Both pre- and post-treatment with CA-4 showed a reduction in DNA damage compared to H_2_O_2_-treated controls, with pre-treatment with 500 nM proving to be significantly more effective than post-treatment with 10 nM and 1 µM concentrations.

## 4. Discussion

In this study, we demonstrated significant differences in sensitivity to CA-4 between healthy, normal cells and cancer cells. CA-4 and its analogues have been shown to exert cytotoxic effects on a range of tumor cells at nanomolar-to-micromolar concentrations, including cells resistant to other antitumor agents [4,5,12]. Previous studies documented the most potent antiproliferative activity of CA-4 against various cancer cell lines, with IC_50_ values ranging from 0.08 to 35.6 µM [25] and from 0.36 to 7.08 µM [26]. These concentrations are well below the threshold of 200 µM, at which we observed sensitivity in primary lymphocytes and normal trophoblast cells (see Figure 1A,B). Consistent with our results, other studies have reported that CA-4 has significantly lower cytotoxicity toward healthy cells compared to tumor cells [25]. However, one study showed lower selectivity, with CA-4 exhibiting antiproliferative effects on human lymphocytes, although it still exhibited significantly higher cytotoxicity for HL-60 (human leukemia) and OVCAR-8 (ovarian adenocarcinoma) tumor cell lines [27]. Taken together, our results, along with previous findings, suggest a clear selectivity of CA-4 cytotoxicity toward tumor cells compared to non-dividing healthy lymphocytes, with proliferating lymphocytes being more sensitive. Healthy trophoblast cells and PBMCs showed at least 100-fold lower sensitivity, which only became apparent at high micromolar doses, while HeLa tumor cells showed significant sensitivity even at concentrations as low as 1 µM (as seen in Figure 1). Although the IC_50_ values obtained for CA-4 in choriocarcinoma cells are relatively high (Figure 1), CA-4 has several unique advantages compared to other herbal compounds and existing chemotherapeutic agents that could make it a useful adjunct therapeutic. CA-4 is classified as a vasodestructive agent. Unlike the conventional chemotherapeutic agents that primarily target the tumor cells, CA-4 disrupts the tumor’s blood vessels, cutting off oxygen and nutrient supply, and leading to rapid necrosis of the central tumor mass. In addition, many herbal agents, such as paclitaxel or vincristine, primarily inhibit cell division (mitosis) by targeting the microtubules. CA-4 binds to tubulin and inhibits microtubule polymerization, preventing cell division. However, CA-4 binds to a different site on tubulin than other agents, such as paclitaxel or vinblastine, called the colchicine-binding site. This unique binding reduces the risk of cross-resistance with other tubulin-targeting agents. CA-4 is often studied in combination therapies because it may enhance the efficacy of other treatments, such as radiation or conventional chemotherapy. However, its use in combination therapies in choriocarcinoma is not yet clear. 

Of particular importance is the relative resistance of lymphocytes to potential antitumor drugs, as they play a crucial role in fighting tumor cells [21,22], which could contribute to a better cure and a better long-term prognosis. In addition, the observed resistance of normal trophoblast cells to CA-4 underscores its potential safety profile during pregnancy. Notably, however, JAR choriocarcinoma cells showed greater resistance to CA-4 compared to cervical carcinoma HeLa cells, although they were still more sensitive than healthy trophoblasts (Figure 1C,D). These results provide initial data on the *in vitro* anticancer potential of CA-4 in human choriocarcinoma and normal trophoblast cells. Future research on the application of CA-4 in the treatment of gestational trophoblastic cancer requires further validation through *in vivo* studies, as well as a thorough an examination of the safety aspects and the molecular mechanisms involved.

In addition, our results suggest that CA-4 exerts an antioxidant and protective effect in healthy lymphocytes (Figure 2). This is consistent with previous studies demonstrating the inherent antioxidant properties of CA-4, with IC_50_ capture values of 4.65 mg/mL and 5.71 mg/mL for its *cis* and *trans* isomers, respectively, compared to the IC_50_ value of ascorbic acid of 8.43 mg/mL [28]. Interestingly, CA-4 has shown opposite effects in cancer cells by significantly increasing ROS levels, thereby exceeding the threshold for oxidative stress. By further increasing ROS production in cancer cells, CA-4 induces oxidative damage, which disrupts important cellular processes and ultimately leads to cell death [4,12,13]. This differential action between normal and cancer cells highlights the selective anticancer effect of CA-4.

Previous studies have also shown that this pro-oxidant effect in cancer cells is accompanied by a reduced activity of important antioxidant enzymes, such as catalase (CAT) and glutathione reductase (GR), while the activity of superoxide dismutase (SOD) remains unchanged. These results suggest that CA-4 interferes with the cellular antioxidant defense system and leads to ROS accumulation and apoptosis at nanomolar concentrations (83, 130, and 190 nM) [29].

Although the exact mechanism by which CA-4 reduces ROS in stimulated lymphocytes remains to be fully elucidated, the differential effects observed between healthy and tumor cells strongly support the selective antitumor activity of the compound. In conjunction with the data on metabolic activity and ROS production, these results emphasize the potential of CA-4 as an anticancer drug, especially given its lack of cytotoxicity in the therapeutic concentration range and its protective effect on lymphocytes under oxidative stress [4,6,14,15,16].

Similar to our findings on the genotoxicity of CA-4 in human PBMCs (Figure 3A), previous studies have not found clastogenicity of CA-4 in human peripheral blood lymphocytes *in vitro*. In these studies, no increase in structural chromosomal aberrations was observed over a wide range of CA-4 concentrations (50 to 500 µg/mL) with or without metabolic activation [30]. Of note, the lowest concentration tested in this study, 50 µg/mL, corresponds to approximately 158 µM, which is much higher than the concentrations used in our comet assay. In contrast, in vivo studies in Swiss mice showed a dose-dependent increase in DNA damage with increasing doses of CA-4 analogs [31]. Another study showed that CA-4P treatment in leukemia/lymphoma cell lines caused mitochondrial membrane disruption, increased intracellular ROS, and DNA fragmentation, ultimately leading to cell death, suggesting that ROS accumulation is a critical factor in CA-4P-induced cytotoxicity [32].

In normal cells, robust antioxidant defense mechanisms and efficient DNA repair systems, such as the base excision repair (BER) pathway, mitigate the effects of DNA damage. However, cancer cells are more susceptible because DNA repair mechanisms are impaired, and the baseline level of oxidative stress is increased [20,24]. Defective DNA repair mechanisms in cancer cells lead to increased constitutive DNA damage [19]. Combretastatins, including CA-4, exacerbate this stress by driving cancer cells beyond their threshold for tolerable DNA damage, leading to apoptosis [4,17,18].

## 5. Conclusions

In this study, combretastatin A-4 (CA-4) exhibited selective cytotoxicity by exerting significant cytotoxic effects on cancer cell lines, while sparing healthy human PBMCs and trophoblast cells. CA-4 showed minimal cytotoxicity in normal cells at therapeutic concentrations, preserving metabolic activity and even reducing oxidative stress-induced DNA damage and ROS levels. These results emphasize the potential of CA-4 as a selective anticancer agent with antioxidant and antigenotoxic properties in normal cells and warrant further investigation into its use in cancer therapy, particularly in trophoblastic cancer.

## Data Availability

Dataset available upon request to the authors. The raw data supporting the conclusions of this article will be made available by the authors upon request.
